# Effectiveness of fetal scalp stimulation test in assessing fetal wellbeing during labor, a retrospective cohort study

**DOI:** 10.1186/s12884-020-03030-7

**Published:** 2020-06-05

**Authors:** Farzaneh Shakouri, Linda Iorizzo, Hellen Mc Kinnon Edwards, Christina Anne Vinter, Karl Kristensen, Per-Erik Isberg, Nana Wiberg

**Affiliations:** 1 Department of Gynecology and Obstetrics, Sjælland University Hospital, Roskilde, Denmark; 2 Department of Clinical Sciences Lund, Lund University, Lund, Sweden; 3 Department of Gynecology and Obstetrics, Helsingborg Hospital, Helsingborg, Sweden; 4 Department of Gynecology and Obstetrics, Herlev and Gentofte Hospital, University of Copenhagen, Copenhagen, Denmark; 5 Department of Gynecology and Obstetrics, Odense University Hospital, Odense, Denmark; 6 Institute of Clinical Research, University of Southern Denmark, Odense, Denmark; 7 Department of Clinical Sciences Lund, Gynecology and Obstetrics, Lund University, Faculty of Medicine, Lund, Sweden; 8 Department of Statistics, Lund University, Lund, Sweden; 9 Department of Gynecology and Obstetrics, Skåne University Hospital, Ystad, Sweden

**Keywords:** Fetal monitoring, Fetal scalp stimulation, Fetal scalp blood lactate, Cardiotocography, Umbilical cord blood, Hypoxia, Acidemia

## Abstract

**Background:**

It is discussed whether fetal scalp stimulation (FSS) test is a reliable complimentary tool to cardiotocography (CTG) to assess fetal wellbeing during labor. The test is based on the assumption that a well-oxygenated fetus, in contrast to the depressed fetus, will respond to a certain stimulus. The aim of this study was to investigate the effectiveness of the FSS-test.

**Methods:**

A retrospective observational study carried out Copenhagen University Hospital, Herlev, Denmark. Laboring women with singleton pregnancies in cephalic presentation after gestation week 33 and indication for fetal blood sampling (FBS) were eligible for inclusion. The FSS-test was classified as positive when an acceleration was absent at the time of FBS and negative when an acceleration was present. Lactate in scalp blood was measured by the point-of-care device LactatePro™ and pH in artery umbilical cord blood by the stationary blood gas analyzer ABL800. Lactate level < 4.2 mmol/L in scalp blood and arterial cord pH > 7.1 were cut-offs for normality.

**Results:**

Three hundred eighty-five women were included. The cohort was divided by the FBS-to-delivery time: Group 1 (*n* = 128) ≤ 20 min, Group 2 (*n* = 117) 21–59 min and Group 3 (*n* = 140) ≥ 60 min. The proportion of FSS-positive tests differed significantly between the groups (*p* < 0.000). In Group 1 the sensitivity, specificity and likelihoods for scalp lactate ≥4.2 mmol/L were 81.5 (95% CI 67–90.1), 13.3 18.5 (95% CI 5.9–24.6), LHR+ 0.94 (95% CI 0.8–1.1) and LHR – 1.4 (95% CI 0.6–3.2) and for umbilical artery pH ≤ 7.10 the values were 82.6% (95% CI 61.2–95.1), 16% (95% CI 9.4–24.7), 1.0 (95% CI 0.8–1.2) and 1.1 (95% CI 0.4–3) respectively. Regardless of the FBS-to-delivery time the LHR+ for lactate ≥4.2 mmol/L increased to 1.38 (95% CI 1.2–1.6).

**Conclusion:**

The effectiveness of scalp stimulation test was poor for both ruling in and out fetal hypoxia during labor. Absence of a provoked acceleration seems to be a normal phenomenon in the second stage of labor.

## Background

Intrapartum fetal surveillance by cardiotocography (CTG) was introduced in the late 1960s, to early recognize and respond to signs of intrapartum fetal distress, attempting to avoid severe intrauterine hypoxia resulting in perinatal deaths or brain/organ damage [1]. CTG is characterized by a high sensitivity, a low specificity and a low positive predictive value for adverse outcomes, but also a high intra- and inter-observer variation potentially leading to an unnecessary and inappropriately high operative delivery rate with risks for both the fetus and the mother [2]. In low-income countries continuous CTG during labor is still a privilege and usually intermittent auscultation is practiced even for high risk pregnancies [3]. To improve the outcome and to reduce interventions various second line tools have been suggested [4]. Fetal scalp stimulation (FSS) test was first described in 1936 by Sonntag and rests on the assumption that a reassuring fetus or a fetus with mild acidemia will respond to a certain stimulus by an increase in the heartrate [5, 6]. Four methods for fetal stimulation are described in the literature: vibroacoustic stimulation, Allis clamp application, digital stimulation or puncture of the scalp for fetal scalp blood sampling (FBS) [7]. The only metanalysis published is based on 11 articles, all studies with a small number of participants for each of the four tests. Exclusively, one article describes the stage of labor when the FSS-test was conducted. Despite this, the authors warrants for the use of the FSS-test argued by the low likelihood ratio for fetal acidemia given a negative test i.e. fetal response at stimulation [7]. Intrapartum FBS with measurement of scalp blood pH or lactate provides additional information of the acid-base status of the fetus and the internationally accepted cut-offs for normality are: pH ≥ 7.25 and lactate < 4.2 mmol/L [8]. From observational studies there is growing evidence for FBS to be associated with decreased operative deliveries and perhaps also a reduction in severe neonatal acidosis [4, 9, 10].

The aim of this study was to investigate the effectiveness of the FSS-test for fetal acidemia defined as a pathological high lactate in scalp blood, and for women with 20 min from FBS to delivery also as a pathological low pH in umbilical cord blood.

## Methods

### Study design

From November 2013 to Maj 2014 a prospective cohort study was conducted at two university hospitals with the aim to propose cut-offs for the handheld lactate-meter Lactate Pro™2 [11]. For the actual study only women from one of the two centers were included (Herlev University Hospital, Copenhagen, Denmark). All women in active labor with an indication for FBS due to a non-reassuring CTG (suspicious or pathological trace) or a significant STAN event (ST-waveform analysis of the fetal electrocardiogram, STAN®, Neoventa Medical, Gothenburg, Sweden) were enrolled if the inclusion criteria was obtained: a fetus in cephalic presentation and > 33 weeks of gestation. In case of multiple pregnancy, breech presentation or risk for vertical transmission the women were excluded. Obstetrical and neonatal data was recorded in the primary study database. The cohort was divided into three groups corresponding to the FBS-to-delivery time, Group 1: ≤ 20 min, Group 2: 21–59 min and Group 3: ≥ 60 min.

In the original study the indication for FBS was based on the CTG interpretation by midwifes and doctors on call [12]. In Denmark the FIGO classification from 1987 is used for CTG classification as recommended by the Danish society of Obstetrics and Gynecology [13, 14]. To classify a CTG tracing at least 20 min readable registration is obligatory. Therefore, we choose to re-assess the CTGs for up to 30 min before FBS in order to define the baseline and for the fetal response (acceleration) to scalp stimulation (wiping and puncture) up to 5 min after FBS. An acceleration could only be defined if the baseline was definable before FBS and if there was an increase in the fetal heart rate by at least 15 beats per minute for more than 15 s [5, 15]. The FSS-test was regarded positive when no acceleration was seen and negative when an acceleration could be identified as described in previous publications on the subject [7]. The CTGs were re-interpreted by one of the authors (F.Z), during November 2017 until June 2018. The appraiser was blinded to the FBS result, delivery mode and neonatal outcome.

### Biochemical analysis

FBS was performed by the doctor on call by the standard technique: carefully wiping/cleaning the skin (takes normally up to 15–30 s), thereafter puncture the scalp to a depth of 1 mm, wiping again, and finally collecting the blood in pre-heparinized capillary tubes containing up to 100 μl. The blood was blown out from the syringe and analyzed bedside by the point-of-care device LactatePro1™ with the result displayed within one minute. The test is based on an amperometric method using an enzymatic reaction and is calibrated for every 25th analysis with a control test strip. Following internationally recommended cut-offs are: a lactate level < 4.2 mmol/L = normal, 4.2–4.8 mmol/L = pre-acidemia and > 4.8 mmol/L = acidemia [16]. According to our guidelines the FBS should be repeated after 20 min if the measured lactate value is ≥4,2 mmol/L and the CTG pattern persists non-reassuring [13, 17]. If lactate is > 4.8 mmol/L immediate delivery is recommended. Umbilical cord blood was sampled from the unclamped cord directly after delivery and analyzed within 15 mins by a stationary blood gas analyzer (ABL 800, Radiometer, Copenhagen).

### Statistical analysis

Ratios were analyzed and compared by Chi-square test or Kruskal-Wallis test. Group comparison of continuous variable was performed with Mann-Whitney U. For scalp lactate the whole cohort was included in the analysis, whereas for the umbilical cord blood gases (UCBG) only women delivered within 60 min from FBS to delivery were included. Crosstabulation was used for calculation of sensitivity, specificity and likelihood ratios. Likelihood ratios are often used to compare the diagnostic value of a test due to their independence of the prevalence. The likelihood ratio is considered significant when the 95% CI does not cross one. A two-tailed *p*-value of less than 0.05 was considered statistically significant. Analyses were performed using SPSS 25.0 (SPSS, Chicago, IL, USA).

### Ethics

The Regional Ethics Committee in Copenhagen, Denmark, regarded the study as a register study and deemed no need for written consent (H-6-2014-FSP-016).

## Results

A total of 438 women were enrolled, 53 excluded (46 due to a missing CTG tracing or a tracing impossible to classify, one because the gestational age was below 33 weeks, and 6 were without registered time at FBS) leaving 385 women for final analysis. The maternal baseline characteristics between the positive and negative FSS-test results are shown in Table 1. Repeated scalp blood sampling from the same fetus was significantly associated to a positive FSS test (*p* < 0.025). There was a trend towards more women having a negative test when the labor course was stimulated by oxytocin (*p* = 0.051).

**Table 1:** Baseline characteristics of the study population (*n* = 385). An acceleration was defined as an increase in fetal heart rate ≥ of 15 beats per minute lasting at least 15 S*. medians* [Range] and number (%)

	No acceleration (Positive test) *n* = 245	Acceleration (Negative test) *n* = 140	*p*-value
Maternal age, years	30 [18–46]	30 [20–42]	0.981
Gestational age, days	282 [232–296]	284 [234–294]	0.432
	n (%)	n (%)	
Induction of labor	62 (25)	39 (28)	0.63
Fever during labor (≥ 38 °C)	54 (22)	23 (17)	0.234
Epidural anesthesia	125 (51)	76 (54)	0.596
Oxytocin augmentation	139 (57)	94 (67)	0.051
Repeated FBS	113 (46)	48 (34)	0.025

*FBS* fetal scalp blood sampling, *CTG* cardiotocography, *SD* Standard derivation. Mann-Whitney for continuous data, Fisher’s exact test for nominal data

After the cohort was divided by the FBS-to-delivery time; there were 128 women in *Group 1* ≤ 20 min, (108 with a positive test, 20 with a negative), 117 women in *Group 2* 21–59 min (69 with a positive test, 48 with a negative) and 140 women in *Group 3* > 60 min (68 with a positive test, 72 with a negative) with significant difference in the number of positive/negative test between the three groups (*p* < 0.000).

In Group 1 93% of women were delivered vaginally. There was no significant difference in level of scalp lactate or UCBG between those with a positive versus a negative FSS test. In contrast, for Group 2, 73.5% were delivered vaginally and for that group there was a significant difference in the level of scalp lactate and arterial umbilical base-excess, Table 2.

**Table 2:** Scalp lactate, UCBG and Apgar score < 7 at 5 min in 385 women with need of FBS during delivery. The cohort is divided by time from FBS to delivery and in positive and negative test results i.e. no versus an acceleration on CTG at FBS. The UCBG are not shown for women with ≥60 min from FBS to delivery. Medians with [range]

	No acceleration (Positive test)	Acceleration (Negative test)	*p* value
**FBS to delivery ≤ 20 min (*n*** **= 128)**	108	20	
Scalp lactate mmol/L	4.2 [1.6–7.7]	4.4 [1.9–7.0]	0.66
pH	7.2 [6.99–7.34]	7.2 [7.06–7.31]	0.790
A-pCO_2_ (kPa)	7.48 [4.68–13.6]	8.12 [5.18–10.2]	0.408
A-BE	−6.8 [−17–0.8]	−6.1 [− 12-(− 2)]	0.829
A-lactate mmol/L	7.5 [3.6–12]	6.3 [4.5–9.1]	0.748
5 min AS < 7	3	0	NA
**FBS to delivery 21–59 min (*n*** **= 117)**	69	48	
Scalp lactate mmol/L	3.2 [1.1–6.8]	2.3 [1.0–6.4]	0.012
pH	7.23 [7.04–7.32]	7.22 [6.99–7.35]	1.00
A-pCO_2_ (kPa)	7.31 [4.38–11]	7.88 [5.09–11.1]	0.472
A-BE	−5.95 [−17–1.4]	−4.15 [−10.8–7.3]	0.044
A-lactate mmol/L	5 [2.6–10.0]	6.5 [2.9–8.9]	0.609
5 min AS < 7	2	0	NA
**FBS to delivery ≥ 60 min (n = 140)**	68	72	
Scalp lactate mmol/L	2.1 [1–5]	2.1 [1.1–4.2]	0.911

*UCBG* umbilical cord blood gases, *FBS* fetal blood sampling, *Min* minutes, *A* artery umbilical cord blood, *pCO*_*2*_ partial pressure of CO_2_, *BE* base excess, NA: not applicable

Mann-Whitney test for continuous data, Fisher’s exact test for nominal data

The sensitivity, specificity, positive likelihood ratio (LHR+) and negative LHR (LHR-) for the FSS-test are shown in Table 3. The sensitivity for scalp lactate ≥4.2 mmol/L and > 4.8 mmol/L was better within 20 min from FBS to delivery if compared to the whole cohort, whereas the specificity was improved for Group 1 for both outcomes. In Group 1 the (LHR+) for prediction of acidemia i.e. lactate ≥4.2 mmol/L or lactate ≥4.8 mmol/L or A-pH ≤ 7.10 given a positive test were: 0.94 (95% CI 0.8–1.1), 0.85 (0.7–1.0) and 0.98 (95% CI 0.8–1.2), respectively. The (LHR-) were for the same parameters 1.38 (95% CI 0.6–3.15), 2.17 (95% CI 0.97–4.84) and 1.09 (95% CI 0.4–2.95), respectively. For the whole cohort see Table 3.

**Table 3:** Effectiveness of fetal blood sampling as a stimulation/diagnostic test for the condition of fetal acidemia i.e. abnormal value in scalp blood or low pH in umbilical artery blood. Lactate in mmol/L

	No acceleration Test positive	+ Acceleration Test negative	Sensitivity % (95% CI)	Specificity % (95% CI)	LHR+ (95% CI)	LHR- (95% CI)
Whole cohort not divided by time from FBS to delivery, n = 385	245	140				
Scalp lactate < 4.2	161	120	79 (69.4–86.6)	42.7 (36.9–48.7)	1.38 (1.19–1.59)	0.49 (0.33–0.74)
Scalp lactate ≥4.2	75	20				
Scalp lactate ≤4.8	194	125	73.7 (60.3–84.5)	39.2 (33.8–44.8)	1.21 (1.01–1.45)	0.67 (0.43–1.06)
Scalp lactate > 4.8	42	15				
FBS to delivery ≤20 min n = 128	108	20				
Scalp lactate < 4.2	52	8	81.5 (67–90.1)	13.3 (5.9–24.6)	0.94 (0.81–1.10)	1.38 (0.61–3.15)
Scalp lactate ≥4.2	53	12				
Scalp lactate ≤4.8	71	9	75.6 (60.5–87.1)	11.3 (5.3–20.3)	0.85 (0.71–1.02)	2.17 (0.97–4.84)
Scalp lactate > 4.8	34	11				
pH ≤ 7.1	19	4	82.6 (61.2–95.1)	16 (9.4–24.7)	0.98 (0.8–1.21)	1.09 (0.4–2.95)
pH > 7.1	84	16				

*FBS* fetal blood sampling, *LR* likelihood ratio, *t* confidence interval

## Discussion

In this study we show that the specificity and LHRs for both ruling in and out fetal acidemia by scalp stimulation test is poor. The LHRs were close to one, except for scalp lactate > 4.8 mmol/L within 20 min from FBS to delivery. Also, the scalp lactate value and the UBCGs were remarkably similar between the fetuses despite a positive or negative test. Due to few cases (*n* = 7) with an Apgar score at 5 min < 7 we were unable to calculate statistics but six of the cases were in the group with a positive test.

Our results differ significantly from most of the published results inclusively the meta-analysis but are very similar to the results found in the study by Holzman et al. [3, 7, 18, 19]. One explanation could be measurements of pH rather than lactate as in Holzmann’s and our study. It is for debate whether pH or lactate should be preferred. A new secondary analysis showed no significant difference in neonatal outcome between the methods despite that there was a trend towards a lower number of pH < 7 and 5-min AS < 7 and 4 in the lactate arm [20].

Traditions and level of clinical experience are affecting the frequency of FBS and potentially thereby also the clinical outcome [21–23]. For this study the decision to complement CTG with FBS was performed by the first doctor on call as described in the primary article [11]. With a normal FBS result although of a non-reassuring CTG the labor is usually allowed to continue in our hospital. Theoretically this can imply a longer labor course eventually with a slightly deterioration of the fetal scalp blood lactate and the UCBGs towards more acidotic values compared to a cohort where FBS is non-prioritized for expedition of labor. This can, theoretically, contribute to the discrepancy in our results explained by the difference in pre- and post-test probabilities [21, 24–26].

CTG is known for its poor specificity potentially leading to unnecessary instrumental delivery. Despite many trials’ researchers have not proven to find a better method or an optimal reliable secondary tool. Many secondary tools are suggested such as for example the FSS-test [15, 27, 28]. FSS-test can be practiced by scalp stimulation, puncture, or Allis clamp application where the two latter methods have been questioned due to the painful procedure. Pain normally results in a decrease in vagal tone (parasympaticus) directly followed by an increase of sympaticus. In case of hypoxia the autonomic nervous system seems to fail, resulting in only activation of parasympaticus [29, 30]. We tried to come across the issue by the gently sweeping/cleaning before puncture. By that we would expect an increase in heartrate before the decrease in vagal tone.

A (LHR+) greater than 10 or a (LHR-) less than 0.1 have the potential to alter clinical decisions [31]. Compared to other studies all based on a small number of cases and mostly with a wide 95% CI we found a considerably low (LHR+) and especially in women delivered shortly after FBS [7, 18]. Our results are in line with the previously mentioned Swedish study from 2016 based on a cohort of 1070 women (indication for FBS due to non-reassuring CTG) where the authors showed a (LHR+) of 1.15 and a (LHR-) of 0.14 for the properties of FBS as FSS-test. In Group 1 93% of the women were delivered vaginally assuming that they were in the second stage of labor at the time when FBS was performed. Except from two studies we were not able to address when in the labor course the FSS/FBS was performed why we analyzed the whole cohort independently of which stage in labor the FBS was performed. In the second stage FBS is normally not recommended and our believe is that most obstetricians expedite delivery if CTG deteriorates during that phase. If the majority of studies are based on performance of the FSS tests during the first stage or early in the second phase before pushing the fetus will be less acidotic compared to the active second stage [24]. Secondly, during the second stage the fetal head and eye bulbuls are exposed to extreme pressure from surrounding tissues. It is likely that the fetus becomes desensitized to pain by an increased release of endorphins and therefore is unable to react to a pain stimulus on its way through the birth canal [32]. Not only pressure on the eye bulbul, but also natural oxytocin and augmentation with artificial oxytocin activates parasympaticus why the absence of accelerations during second stage is an unspecific sign not related to hypoxia [4, 15, 18, 33–35]. Twenty fetuses in our study had the ability to generate an acceleration despite a diagnosed acidemia in scalp blood. Theoretically this can be explained by an extraordinary release of the stress hormones through the stimulus of sympaticus or a lower concentration of natural oxytocin and that adrenalin/nor-adrenalin mitigates the effect of parasympaticus. With this in mind, it is important to remember that even mild hypoxia has been associated to impaired childhood outcome, the reason for why we chose the cut-off for pre-acidemia defined by scalp lactate ≥4.2 mmol/L [17, 36, 37].

We cannot exclude that FSS test is an alternative to FBS in first stage of labor. We saw considerable changes in the specificity and LHRs when we compared the group with short time from FBS to delivery with the whole cohort although the specificity and LHR+ was very low implying the risk of unnecessary expedition of delivery. The sensitivity was good but not impressive, potentially leading to missing of depressed fetuses. In a totally different setting as in low income countries with no availability for CTG and a high incidence of intrapartum acidosis the FSS-test recently showed promising results i.e. reduction of newborns born with acidosis when used as a complimentary tool to doppler auscultation [3]. However, it would be a mistake to compare such a setting to settings in the developed countries.

### Strengths

The major strength of this study is the inclusion of 385 consecutive deliveries systematically recorded. According to our guideline continuous CTG is always used in risk pregnancies, in cases with abnormal doppler auscultation during the first stage of labor and for all women in second stage of labor. By routine, cord blood is analyzed in all newborns and not only after risk pregnancies or deliveries.

### Limitations

Severe hypoxia is a rare outcome. Due to our study size the results would need to be confirmed in a larger study.

## Conclusion

There is an association between the fetal ability to react to a scalp stimulus and the fetal metabolism. However, we found the efficiency of FSS test too poor to rule in or rule out fetal hypoxia. Therefore, we recommend using the FSS test with caution, especially during the second stage where absence of accelerations also after provocation seems to be a normal phenomenon.

## Data Availability

The datasets used are available from the corresponding author on reasonable request.

## Abbreviations

FSS: Fetal scalp stimulation test
CTG: Cardiotocography
FBS: Fetal blood sampling
UCBG: Umbilical cord blood gases
LHR +: Positive likelihood ratio
LHR-: Negative likelihood ratio
